# Diabetes and Driving: How Predictive Technologies Will Alter Ethical and Legal Responsibilities

**DOI:** 10.1177/19322968261468763

**Published:** 2026-09-06

**Authors:** Sinead Prince, Jamie Walvisch, Matthew L. Baum, Tasmia Hai, Danièle Pacaud, Julian Savulescu, Fergus Cameron

**Affiliations:** 1 Centre for Biomedical Ethics, Yong Loo Lin School of Medicine, National University of Singapore, Singapore; 2 UWA Law School, The University of Western Australia, Perth, Australia; 3 Department of Psychiatry, Brigham and Women’s Hospital, Boston, MA, USA; 4 Center for Bioethics, Harvard Medical School, Harvard University, Boston, MA, USA; 5 Children’s Hospital Research Institute of Manitoba, University of Manitoba, Winnipeg, Canada; 6 Department of Educational and Counselling Psychology, McGill University, Montréal, QC, Canada; 7 Department of Pediatrics, Cumming School of Medicine, University of Calgary, AB, Canada; 8 Murdoch Children’s Research Institute, Melbourne, VIC, Australia; 9 Uehiro Oxford Institute, University of Oxford, UK; 10 The Royal Children’s Hospital, Melbourne, VIC, Australia

**Keywords:** bioprediction, driving, ethics, hypoglycemia, law, responsibility

## Abstract

In 2023, a 67-year-old man with long-standing type 1 diabetes experienced a severe hypoglycemic episode and drove into an outdoor dining area in Victoria, Australia, killing 5 people and injuring several others. Although the charges against the driver were dismissed, future cases involving new continuous glucose monitors enhanced with artificial intelligence that allow users to anticipate impairment before it occurs may have different outcomes. These biopredictive technologies transform hypoglycemia from a background risk into a more foreseeable event. This generates new conditional obligations to act on predictive warnings and, in some cases, obligations on those with higher risks to acquire such technologies. Furthermore, these individual responsibilities can generate corresponding state obligations to subsidize access and update licensing frameworks, ensuring safety without exacerbating inequity or stigma.

## Introduction

In 2023, a 67-year-old man with long-standing type 1 diabetes experienced a severe hypoglycemic episode and drove into an outdoor dining area at the Royal Daylesford Hotel in Victoria, Australia, killing 5 people and injuring several others.^1^ The court scrutinized a sequence of warnings from his continuous glucose monitor (CGM) and opportunities for intervention preceding the collision. The driver had tested his blood glucose at 3:58 PM with a stable reading of 7.2 mmol/L. However, between 4:57 PM and 5:17 PM, his CGM recorded 5 “Signal Loss Alarms,” indicating that the monitor had stopped communicating with his phone for a 40-minute period. When monitoring resumed, a manual check at 5:17 PM revealed a critically low reading of 2.9 mmol/L, followed shortly after by a “hypoglycemia alarm” and “low glucose” notification. The man attempted to purchase food from a deli but was turned away due to full venue capacity. He resumed driving at 5:36 PM and crashed approximately 30 minutes later.

The prosecution argued that the man was grossly negligent by ignoring the information conveyed by the signal loss warnings, the low reading, and the subsequent alarms. The defense, however, successfully contended that he lacked the capacity for voluntary action at the relevant time. Experts testified that due to the physiological lag in interstitial readings, the 2.9 mmol/L result likely reflected his status from 5 to 15 minutes prior, suggesting he already lacked capacity when the alarms sounded. The magistrate ultimately dismissed all 14 charges, finding the prosecution could not exclude the hypothesis that he was acting involuntarily (in a state of automatism) when he commenced the final, fatal drive. Currently, a coronial inquest is being held in Victoria to examine the “guidelines, education, and public awareness” for drivers with diabetes and “outdoor dining and road safety.”^2^

While we might wonder whether this tragedy could have been avoided if the driver had consumed the fast-acting carbohydrates seemingly available in his car,^3^ this case raises broader ethical questions about the responsibilities of those using diabetes management devices that can generate advance warnings. How will responsibilities change when artificial intelligence (AI) improves the timeliness, quality, and reliability of device warnings and, in some cases, automates interventions well before impairment sets in.^4-9^ Although AI raises a host of ethical issues for diabetes management and driving (see Table 1), we focus on whether drivers have an obligation to act upon and acquire predictive warnings of hypoglycemia, whether use of such technologies might become a prerequisite for obtaining a driving license, and whether there are corresponding state obligations to ensure such mandates do not exacerbate inequity.

**Table 1.** A Summary of Existing Ethical Issues Regarding the Development, Integration, and Regulation of AI in Diabetes Management and Driving.

Ethical issue	Explanation	Example in the context of diabetes and driving
*Explainability and transparency* ^10^	Some AI models have “black box” or opaque systems that result in outputs or recommendations being unexplainable. This results in concerns around transparency and concerns about hidden errors.	If a CGM advises someone with diabetes that they are safe to drive even though the person feels unsafe, if the system does not show which sensor readings or patterns triggered the alert, the driver may not know whether to trust it or how to respond appropriately.
*Bias and fairness* ^11^	AI requires significant amounts of data for training and algorithm development. Historically, health care data poorly represents underprivileged groups leading to AI outputs that may exacerbate existing bias and contribute to further inequalities in health care outcomes.	If predictive CGMs are trained on predominantly male glucose data, these systems may not work safely for females, thus increasing their risk of hypoglycemia and, consequently, driving risks.
*Informed consent and user understanding* ^12^	In certain circumstances, the use of AI may undermine patient autonomy, whether because patients do not know that AI is used in their care, whether their data are used, the potential risks and limitations of AI capabilities, and whether the black box conceals essential information for informed consent.	A driver might enable “safety mode” in a navigation app without realizing that the app continuously analyses glucose data and may automatically report “unsafe driving while hypoglycemic” to third parties, limiting genuine consent.
*Safety and reliability* ^13^	If AI is not regulated through health regulatory systems, AI tools might not be sufficiently validated for health care use. If AI is deployed outside the community on whose data it was trained, it may not be sufficiently reliable or accurate for use; developers must be transparent about the population or demographic for whom the AI is safe and reliable and ensure that the AI outputs are monitored over time and integrated with human oversight so that errors are detected and mitigated.	False negatives from an algorithm that predicts imminent hypoglycemia while driving, using CGM trends and past events, could lead to accidents, while frequent false positives could cause unnecessary emergency stops or anxiety.
*Accountability and responsibility* ^14^	When AI makes mistakes or causes harm to a patient, difficulties arise as to who is responsible and how to appropriately allocate responsibility between developers, vendors, clinicians, regulatory bodies, or institutions.	If an AI system incorrectly assures a driver with diabetes that their risk of hypoglycemia is low and they subsequently cause an accident, it may be unclear whether responsibility lies with the driver, the software developer, the device manufacturer, or the health care team that recommended the tool.
*Professional integrity and role of clinicians* ^15^	If physicians are not sufficiently protected by law or regulation in reaching their own judgments, AI may systemically reshape clinical judgment, potentially creating overreliance on automated outputs and eroding the clinician’s role. Furthermore, overreliance on AI-mediated, data-driven interactions may reduce time for relational care, empathy, and narrative understanding that are essential in understanding the patient’s health in the wider context of their lives.	If clinicians start relying on AI-generated “fitness to drive” scores when completing medical driving reports, they might potentially overlook individual psychosocial factors or context (eg, variable work shifts, cognitive load) that the algorithm does not capture.
*Health equity and access* ^16^	While there are hopes that AI tools can narrow health care access disparities to those traditionally excluded or limited in access to health care, there are also concerns that such claims depend on whether the tools are designed to address issues causing health care inequalities, whether the tools will undermine access to human care, and also depend on infrastructure, digital literacy, and cost.	If regulations impose blanket restrictions on all people with diabetes, but only some can afford advanced CGM devices, smartphones, or connected vehicles that provide AI-based driving safety alerts, those without access may be unfairly denied the ability to drive and thus to meet essential needs, even when they present lower actual risk.
*Confidentiality and privacy* ^17^	AI uses big data to develop algorithms and patient data to recommend outputs, and concerns arise as to how training data and patient data are stored, protected, and used in ways that protect user confidentiality and privacy while not hindering improving performance, access to health care, and data quality.	Driving behavior, GPS location, and glucose data transmitted from a vehicle or smartphone to insurers or app providers could be stored or shared in ways that reveal a person’s diabetes status and daily patterns, potentially affecting insurance premiums or employment.
*Regulatory compliance and oversight* ^18^	Ensuring that AI systems comply with medical device regulation, data protection law, and emerging AI-specific rules and that oversight keeps pace with innovation.	An app that effectively functions as a medical device for assessing driving fitness might bypass formal approval, creating uncertainty about whether regulators, transport authorities, or health agencies are overseeing its safety and use in licensing decisions.
*Environmental and resource impacts* ^19^	Big data storage requires significant energy use, infrastructure, and environmental resources leading to concerns about lifecycle impacts of large-scale AI in health systems and whether AI outcomes can be achieved in other, less environment-demanding, ways.	Given 1 in 10 people have diabetes, CGM data for large populations of drivers with diabetes could increase energy use and infrastructure costs, raising questions about whether these systems are the best use of limited digital health resources.

## Diabetes, Driving, and Bioprediction

Diabetes carries a modestly increased risk (12-19%^20^) of motor vehicle accidents, primarily due to acute hypoglycemia (defined as a blood glucose level below 4.0 mmol/L^21^) and impaired awareness of hypoglycemia.^22^ Hypoglycemia severity depends on symptoms: moderate hypoglycemia allows for self-treatment, whereas severe hypoglycemia involves neuroglycopenia (eg, confusion, loss of consciousness) and requires external assistance.^21^ Because executive function, information processing, attention, memory, and visuospatial skills all depend on adequate glucose,^23-25^ even moderate episodes can compromise a driver’s ability to track road conditions, coordinate hand and eye movements, and make timely decisions.^26^

Responsibility for determining fitness to drive has traditionally fallen to clinicians,^26-28^ with drivers expected to follow regulatory guidelines and medical advice (see Table 2). However, research suggests that compliance is inconsistent. Many drivers do not disclose their insulin use,^29^ do not test prior to driving,^30,31^ do not wait the recommended recovery time,^22^ drive while experiencing low blood glucose,^32,33^ fail to stop driving after noticing low glucose levels,^25^ and do not carry or use emergency glucose supplies.^34^ Little evidence exists on the impact of CGMs in reducing these behaviors. One randomized crossover trial in Japan found that drivers receiving CGM alerts while driving spent less time below range than those not receiving alerts (19% compared with 33% of driving time).^35^ One survey found that drivers using real-time CGM were 3 times more likely to detect low blood glucose, but only 1 in 5 had adjusted their alert thresholds for driving.^36^ They were also far more likely to pull over because of suspected low blood glucose, yet more likely to continue driving, and at least half reported difficulties using real-time CGM while driving.^36^

**Table 2.** Description of Hypoglycemia-Related Conditions on Persons With Diabetes Who Intend or are Driving, the Conditions on the Licenses of Persons With Diabetes Who are Being Differentially Treated With and Without Insulin, and Conditions on Those With Commercial Licenses.

Country	Conditions of driving	Conditions of acquiring private license		Special conditions for commercial drivers
		Treated without insulin	Treated with insulin	
Australia^a,37^	• Check BGL before driving. • Do not drive if BGL <5.0 mmol/L. • Wait 30 minutes after eating to drive.	Unconditional license unavailable if: • End-organ complications that may affect driving are present; or • Recent severe hypoglycemic event has occurred. Conditional license may be issued if: • Periodic review is undertaken; and • Treating doctor confirms: ○ End-organ complications are satisfactorily treated; ○ The person follows a regimen that minimizes hypoglycemia risk; ○ The person has hypoglycemia awareness or a documented management plan; and ○ Any recent severe event has been satisfactorily managed.	Cannot obtain an unconditional license. Conditional license may be issued if: • Review every 2 years; and • Treating doctor confirms: ○ No end-organ effects relevant to driving; ○ No recent severe hypoglycemic event; ○ The person follows a regimen that minimizes hypoglycemia risk; and ○ The person has hypoglycemia awareness or a documented management plan.	Non-insulin-treated drivers: unconditional license unavailable. All drivers: Conditional license may be issued if: • Review every year; and • An endocrinologist or diabetes specialist confirms that the driver meets the same conditions required for private drivers and has no end-organ effects that may affect driving.
United Kingdom^38^	• Check BGL before driving and every 2 hours while driving. • Do not drive if BGL <4.0 mmol/L. • Eat if BGL <5.0 mmol/L and wait 45 minutes. • Use finger-stick testing to confirm BGL >5.0 mmol/L before resuming driving.	License revoked if: • A severe hypoglycemic event occurs while driving (revoked immediately); or • More than 1 severe hypoglycemic event has occurred in the past 12 months while the driver was awake; or • The driver has hypoglycemia unawareness. The license may be reinstated if a doctor or specialist confirms that awareness has been regained (RT-CGM alarms cannot substitute for hypoglycemia awareness).	A restricted 1-3 year license may be issued if: • No more than 2 severe hypoglycemic events have occurred in the past 12 months while the driver was awake, and the most recent event was more than 3 months ago; • Driver has adequate hypoglycemia awareness. The license may be reinstated if a doctor or specialist confirms that awareness has been regained (RT-CGM alarms on devices cannot substitute for hypoglycemia awareness) and that the person is not regarded as a likely risk to the public while driving.	Group 2 (bus and lorry) license holders must use a blood glucose meter that stores 4 weeks of data, and will have license revoked if: • A severe hypoglycemic event has occurred in the past 12 months; or • The driver has hypoglycemia unawareness. Licenses can be restored upon satisfaction of all criteria for 1 year.
Japan^39^		License may be denied, suspended, or revoked if the person: • Has hypoglycemia unawareness (unless blood glucose levels can be artificially regulated); • Fails to disclose diabetes on license application; or • Is reported unfit by a physician.	License may be denied, suspended, or revoked if the person: • Has hypoglycemia unawareness (unless blood sugar can be artificially regulated); • Fails to disclose diabetes on license application; or • Is reported unfit by a physician. License may be reinstated after demonstrating improved risk management.	
Denmark^40^		A 5-year license may be issued if: • Physician certifies fit to drive; and • No hypoglycemia for 2 years. License may be revoked or refused if hypoglycemia risk is unstable.	A 2-year license may be issued if: • Physician certifies fit to drive; and • No hypoglycemia for 2 years. License may be revoked or refused if hypoglycemia risk is unstable.	Non-insulin-treated: 2-year license limit. Insulin-treated: Only granted in exceptional cases with public health department approval.
The Netherlands^40^		A 5-year license may be issued if glycemic control is satisfactory and complications are absent.	A 5-year license may be issued if glycemic control is satisfactory and complications are absent.	Insulin-treated: A 3-year license may be granted in exceptional cases. A specialist must approve that glycemic control is satisfactory and complications are absent, and the driver must self-monitor.
New Zealand^41^	• BGL must be >4.2 mmol/L before driving. • Check BGL every 2-3 hours or when symptomatic. • Waiting times before driving after hypoglycemic episode: ○ Mild episode: 1 hour; ○ Severe episode away from driving: 24 hours; ○ Severe episode whilst driving: 4 weeks and specialist review.	Diabetes must be declared on the license application form. A conditional license may be issued if a health practitioner certifies that the person is fit to drive, taking into account their: ○ Risk of hypoglycemia; ○ Hypoglycemic awareness; and ○ Self-monitoring practices.	Diabetes must be declared on the license application form. A conditional license may be issued if a health practitioner certifies that the person is fit to drive, taking into account their: ○ Risk of hypoglycemia; ○ Hypoglycemic awareness; and ○ Self-monitoring practices.	People with insulin-dependent diabetes are not normally allowed to drive taxis, heavy trade, and passenger service vehicles. Exceptions might be made if a diabetes specialist requests one from the Chief Medical Advisor of the NZ Transport Agency.
Canada^b,42^	• Check BGL before driving and every 4 hours while driving or wear a real-time CGM device while driving. • Do not drive if BGL <4.0 mmol/L. • If BG <4.0, wait at least 40 minutes after correcting a hypoglycemia to at least 5.0 mml/L before driving. • Stop driving if experiencing symptoms of severe hypoglycemia and report to health care providers within 72 hours.	Diabetes must be declared on the license application form. A medical assessment may be required depending on the province.^c^ Mandatory reporting by health care providers of individuals treated with insulin secretagogues who have experienced: ○ Any episode of severe hypoglycemia while driving in the past 12 months. ○ More than 1 episode of severe hypoglycemia while awake but not driving in the past 6 months for private (non-commercial) drivers, and in the past 12 months for commercial drivers.	Diabetes must be declared on the license application form. A medical assessment may be required depending on the province.^d^ Mandatory reporting by health care providers of individuals with: ○ Any episode of severe hypoglycemia while driving in the past 12 months. ○ More than 1 episode of severe hypoglycemia while awake but not driving in the past 6 months for private (non-commercial) drivers, and in the past 12 months for commercial drivers.	In addition to the preceding requirements, commercial drivers should undergo a medical examination at the time of application for a commercial license.

a All Australian states and territories apply the *Assessing Fitness to Drive* guidelines, although implementation requirements vary. For example, Victoria requires an endocrinologist to complete the medical assessment, whereas other jurisdictions permit a general practitioner to do so.

b These guidelines come from the Canadian Medical Association and Diabetes Canada; each province has its own requirements.

c 10 out of 13 provinces and territories have mandatory reporting by health care professionals of individuals medically unfit to drive. The frequency of repeat assessment is also variable between provinces.

d See above.

Although finger sticks and real-time CGM data offer accurate snapshots of current blood glucose levels, they inherently require contemporaneous cognitive capacity to interpret and respond. Criminal responsibility ordinarily presupposes that the accused acted voluntarily – that the relevant conduct was a willed, conscious act.^49-57^ Where a severe hypoglycemic episode deprives a driver of conscious control over their bodily movements, the conduct may be involuntary (a state described as automatism), and the prosecution may be unable to prove the voluntary act that the offense requires; the long-recognized intersection of hypoglycemia and criminal responsibility has been discussed elsewhere.^53^ As the Daylesford case illustrates, when harm arises from a hypoglycemic episode, the driver’s conduct at the time of the collision may be found involuntary, so that responsibility cannot attach to those immediate actions. Responsibility will generally need to be traced to an earlier point in time, when the driver was still acting voluntarily, could foresee a risk of harm, and failed to take reasonable steps to prevent it.^54^ This creates a critical vulnerability: because cognitive impairment often begins before a person subjectively realizes it,^31^ a driver may lose the capacity to process or act appropriately on the very data intended to warn them before they appreciate the need to do so. Ordinary real-time CGM data may not, by itself, overcome this difficulty where warnings arrive too late or where the driver no longer has the cognitive capacity to interpret and act on them.

Bioprediction – the use of biomedical data to predict the likelihood of specific physiological states occurring – is different. Within the context of diabetes, this includes CGM systems that use AI and machine learning to analyze glucose trends and physiological data, help users to proactively manage their blood glucose levels, and warn users of impending hypoglycemia well in advance of impairment.^4-9^ Because these predictions are built on CGM data, their reliability is partly constrained by the accuracy of the underlying sensor, which is generally high in current devices^55^ but harder to characterize in the hypoglycemic range most relevant to driving safety.^56^ Emerging evidence suggests that CGM-driven machine-learning algorithms can predict hypoglycemic episodes with moderate overall reliability.^4,5,57^ A systematic review and meta-analysis by Quader et al^58^ found a pooled sensitivity of 80% and a pooled specificity of 89% across 20 studies, with some algorithms reporting sensitivities and specificities up to 99% and prediction horizons of up to 1 hour. Some research has found that explainable machine-learning systems can predict hypoglycemia over the following week with an 89% success rate.^59,60^ Current limitations include variability in false-positive rates,^61^ differences in acceptable false-positive rates between high-risk and low-risk individuals,^58^ and the fact that many algorithms do not account for clinically informative inputs such as insulin dose, meals, and activity.^62^ Furthermore, apart from studies showing that predictive alerts reduce time spent in hypoglycemia generally, there is no research on how biopredictive CGMs affect driver behavior.

## Responsibility to Use Bioprediction

In providing notice to users when they still retain the decision-making capacity necessary to take proportionate precautions, bioprediction changes the moral and legal landscape of responsibility. Moral responsibility traditionally requires meeting two conditions: (1) awareness (the person knew, or should have known, the consequences of their actions) and (2) control (the person had voluntary control over the action).^63^ Historically, severe hypoglycemia excuses responsibility by undermining both conditions: it induces cognitive failure that renders actions involuntary, often with insufficient warning to allow for preventive control.

However, biopredictive technologies enable both criteria of responsibility to be met for acts committed involuntarily, because they provide an earlier opportunity to act when both awareness and voluntary control are present.^64-66^ A person may therefore be liable where they were unimpaired and had a reasonable opportunity to foresee the likelihood of becoming incapacitated but failed to take reasonable steps to avoid the likely consequences of that impairment. This omission could arise across three key moments for people with diabetes when driving: (1) failing to check predictions about blood glucose levels before driving; (2) ignoring a present low-glucose alert by failing to safely cease driving and self-treat; and (3) disregarding a predictive alert of impending hypoglycemia. These failures may constitute a culpable omission that allows liability to be traced to the subsequent harm.

These responsibilities are likely to become increasingly relevant in criminal and civil law cases as biopredictive technologies improve. Courts already have established systems for identifying prior fault where loss of capacity was foreseeable and preventable.^67^ Thus, while courts have struggled to use standard CGM data as evidence of culpable driving, biopredictive technologies may provide increasingly granular evidence of antecedent risk awareness and may consequently assist in determining liability for harm resulting from hypoglycemia-induced cognitive impairment.

## Responsibility to Acquire Predictive Continuous Glucose Monitors

Does the duty to *use* biopredictive technologies extend to all people with diabetes to *acquire* biopredictive technologies to be deemed fit to drive? We argue against a universal mandate now. Biopredictive CGMs are currently expensive;^43,68^ the average cost for these devices is between AUD $3000 and $5000 or USD $6800,^44^ although these figures vary widely depending on country, public health access, and insurance policies. Furthermore, individual risk levels for hypoglycemia vary significantly: about 20% of insulin users account for 80% of severe hypoglycemic events.^45,46^ Imposing such costs might risk converting a public safety objective into an economic barrier to licensure, and requiring all people receiving insulin therapy to acquire such technologies may not be fair.

One way to differentiate between risks is through risk-banding models, in which the strength of obligation to avoid the risk is proportionate to the magnitude and foreseeability of the risk.^47^ Using manual or automated screening, clear criteria, and transparent review pathways, drivers with diabetes could be stratified into high- and low-risk groups.^45,46^

For individuals with high-risk features, acquiring predictive technologies may be the only effective means to ensure safety, particularly when voluntary compliance with existing regulation is unreliable in practice. For example, when a driver cannot reliably perceive the onset of hypoglycemia due to impaired awareness of hypoglycemia, the usual behavioral safeguards may fail even with good intentions. These drivers may therefore have a moral obligation to acquire predictive systems as a condition of licensure when: (1) the risk of severe hypoglycemia is elevated and reasonably foreseeable; (2) non-technological precautions are unlikely to be sufficient; and (3) the relevant technologies are evidence-based, practically available, and financially accessible.

For lower-risk situations, the position is more complicated. New AI tools can predict severe hypoglycemia even for people whose risk is not otherwise apparent.^46,48,59^ However, foreseeability alone cannot ground obligation: in a world of pervasive bioprediction, nearly everyone carries a “non-zero” risk of countless harms.^47^ Moral obligations depend on whether the risk of driving is proportionate to the burdens that might arise in addressing it, including public transport availability, the costs of forgoing driving, and device costs. Given the disproportionality between the current high cost of predictive technologies and the lower probability of harm for lower-risk cases, it is unlikely that we can *presumptively* hold this group responsible for acquiring these tools.

Predictive technologies clearly create responsibilities for users proportionate to the risk of a hypoglycemic episode when driving. They also create new ways for users to satisfy their duties to protect others from these risks. Fully automated closed-loop insulin delivery systems that couple AI-driven prediction with automated delivery allow users to delegate some responsibility for identifying and responding to forecasts of low blood glucose to an automated process.^6^ Users would still be required to maintain the device, respond to alerts, and remain within the limits of the system, but these technologies may mediate responsibility for addressing risk among people with diabetes.

## Responsibilities to Regulate and Subsidize Biopredictive Continuous Glucose Monitors

Most jurisdictions already impose legislative requirements on individuals with diabetes in order to acquire driver’s licenses, such as requiring drivers to monitor glucose prior to driving and to manage hypoglycemia risk in accordance with medical and licensing guidance (see Table 2). In light of predictive technologies and the capacity to screen between risk groups, regulatory bodies ought to adopt a proportionate, risk-based approach: low-risk drivers ought not to be burdened with the obligations designed for higher-risk profiles.

If states make the acquisition of predictive CGMs a condition of licensure for high-risk individuals, they are obliged to ensure access as a matter of justice.^69,70^ The duty to subsidize arises where the cost of compliance would otherwise effectively exclude individuals from licensure.^71,72^ Because driving is often a prerequisite for employment and access to basic goods, especially in rural and suburban regions with poor public transport, states must subsidize these devices to prevent safety regulations from deepening inequality.^72^ This ensures that high-risk drivers can meet moral and licensing requirements without facing unfair burdens.^47^

Policymakers should also ensure that any regulatory scheme does not reinforce stigma toward people with diabetes.^72^ Public health messaging has long portrayed diabetes, particularly type 2 diabetes, as the result of poor self-control, implying that people with diabetes are to blame for their illness.^73^ As such, any regulatory requirements must be accompanied by a careful explanation of their role in reducing identifiable, foreseeable, and preventable harm to others, in ways equivalent to regulations for other medical conditions, such as epilepsy. Regulatory schemes must ensure that expectations placed on drivers are proportionate, realistically achievable, and accompanied by adequate training and assistance.

## Conclusion

Biopredictive technologies are fundamentally reshaping the responsibilities of driving with diabetes. By transforming hypoglycemia from a vague background risk into a foreseeable event, these tools allow moral and legal responsibility to be traced back to earlier omissions.

Consequently, future cases like Daylesford may have different legal outcomes. Drivers with high-risk profiles, particularly those with impaired awareness, might no longer be able to claim that impairment was unpredictable if technology offers a reliable and timely forecast. For this group, the privilege of driving must be tethered to the responsibility to use, and in some cases acquire, these predictive capacities.

However, this new burden is not solitary. As states heighten the obligations on drivers to manage their biology, they are also obliged to enable them to do so. Only through a framework of shared responsibility, where reliable prediction is mandated, accessible and supported, can we ensure that the roads are safe without unjustly penalizing those living with unavoidable medical risk.
